# Factor Structure and Longitudinal Measurement Invariance of the Tangney's Brief Self-Control Scale in Chinese Adolescents

**DOI:** 10.3389/fpubh.2022.802448

**Published:** 2022-03-08

**Authors:** Wei Chen, Guyin Zhang, Xue Tian, Shouying Zhao

**Affiliations:** ^1^School of Psychology, Guizhou Normal University, Guiyang, China; ^2^Center for Big Data Research in Psychology, Guizhou Normal University, Guiyang, China

**Keywords:** self-control, psychometric properties, factor structure, longitudinal measurement invariance, Tangney's Brief Self-Control Scale

## Abstract

Self-control is an important trait for humans to perceive inner and outer perceptions while maintaining harmony with others in society. People with lower self-control are more likely to engage in undesired or irresponsible behavior. The Brief Self-Control Scale (BSCS) is an effective scale with a brief set of items which can effectively measure the level of an individual's control abilities. So far, it has been widely used in many longitudinal studies. However, the factor structure of the scale remains controversial, and far fewer studies have examined the longitudinal measurement invariance of the BSCS. This study aimed to revise the BSCS and test its factor structure for use in Chinese adolescents. Three samples of adolescents (*N* = 1,330/1,000/600, 11–19 years of age) were used. The item-total correlation and inter-item correlation coefficients were used to evaluate the quality of items. The exploratory factor analysis (EFA), confirmatory factor analysis (CFA) and the principle component analysis (PCA) of the residuals were performed to test the factor structure of the BSCS. Three nested models were used to test the longitudinal measurement invariance (LMI) of the BSCS. Pearson correlation coefficient and Cronbach's alpha coefficient were conducted to test the criterion validity and internal consistency reliability, respectively. According to the CFA of different dimensional models of the BSCS, the results did not support the two-dimensional model, and poor factor loading was found for Item 12. Based on this, combined with lower item-total correlation and item-item correlations, Item 12 was eliminated. Based on results of the EFA with both Kaiser eigenvalues and minimum average partial correlations, only one factor of the revised 12-item BSCS was extracted to make the fit indices of the confirmatory factor analysis acceptable. Meanwhile, the results of principle component analysis of the residuals supported the unidimensional assumption. The fit indices of three nested models supported the longitudinal measurement invariance, indicating that this scale has the same meaning over time. The internal consistency coefficient of the BSCS-12 was 0.81 and the test-retest reliability was 0.70. Good concurrent validity was also demonstrated. Overall, these findings suggest that the revised 12-item Tangney's Brief Self-Control Scale has a one-dimensional structure and has good reliability and validity in Chinese adolescents.

## Introduction

Self-control is considered to be an ability to overcome or alter one's dominant responses and to restrain one's undesired behavioral tendencies and avoid acting on them (1). Self-control is an important trait for humans to perceive inner and outer perceptions while maintaining harmony with others in society (1), and is related to self-esteem (2), anxiety (3), thought control, emotional regulation, and impulse inhibition (4). Empirically research has shown that people with high self-control are better at adapting to different situations, are more accommodating in close relationships, and may have a higher sense of wellbeing (4, 5). Conversely, low self-control is a key to predicting delinquency and even serious antisocial behavior (6).

In recent years, juvenile delinquency has occurred frequently and has attracted more attention to the psychological development of adolescents. It has been proven that children with poor self-control show more problem behaviors, such as alcohol use, drug abuse, or teenage pregnancy (7–9). Because of a lack of tolerance when facing frustrations and a feeling of indifference toward others, those with lower self-control are more likely to engage in undesired or irresponsible behaviors (10). Therefore, an individual's ability to maintain self-control is closely related to them exhibiting problem behaviors, and a lack of self-control may lead to criminal behaviors in later adolescence (11). Consequently, it is important to examine self-control in adolescence.

### Measurement of Self-Control

To measure individual trait self-control ability, Tangney et al. compiled the Self-Control Scale (1), which was based on previous theories and empirical literature on self-control. Although the original scale is one of the most widely used measurements in the world and shows good psychometric performance in many countries and regions (4, 12, 13), it has a large numbers of items (36 items) and a intricate factor structure containing five dimensions (i.e., general capacity for self-discipline, deliberate/non-impulsive action, healthy habits, work ethics, and reliability). Notably, it was clearly pointed out that 13 items in the original scale could be combined into a single dimensional structure of the Brief Self-Control Scale (BSCS) in the study of Tangney et al. (1). Regrettably, the related measurement indicators of the BSCS were not reported in their study.

### Factor Structure of the BSCS

Recently, Bertrams et al. (14) found that the German version of the BSCS had a good reliability and validity as a one-dimensional structure, using samples of college students and middle school students. Meanwhile, Brevers et al. (15) used the scale in French speakers and found that the potential structure of the BSCS in French is also unidimensional. However, this result is not consistent with the findings regarding the application of the BSCS in the Netherlands, the United States, or Turkey (16–19), where studies have tended to support the two-dimensional structure. However, these two factors are defined as restraint and impulsivity, with four items, respectively (18); inhibition (six items) and initiation (four items) (16); self-discipline (nine items) and impulse control (four items) (17); and self-discipline (four items) and impulsivity (five items) (19). Recently, Fung et al. (20) found that the two-factor structure was not applicable to their study by using a sample of university students in mainland China. They proposed an 11-item version with four-factor structure, namely: self-discipline (four items), impulsivity (three items), healthy habits (two items) and self-regulation (two items). It's worth noting that although the two-dimensional structure has gained support in different studies and samples, items that could not be classified in their models were discarded, and this factor structure could not be replicated in some studies. In other words, the two-dimensional structure of the BSCS was unstable.

### Longitudinal Study of Self-Control

According to the general theory of low self-control (21), which was proposed to explain all kinds of crimes at all times, stability postulate is a key fact which assumes that the individual's self-control is constant over time. In other words, children with appropriate behavior continue to do well in later adolescence and adulthood, whereas children with low self-control will not be able to control their impulses in the future. However, with the further development of the research, others put forward a new dynamic view of self-control. They liken the stability of self-control to other personality traits, considered that it is not so stable and may change over time (22, 23). Afterwards, more studies were focused on the development of self-control and the BSCS was widely used in many longitudinal studies. For example, Ng-Knight et al. (24) adopted the BSCS in a 3-wave longitudinal study and found that changes of children's self-control were associated with their subsequent performance in secondary school. In addition, Holding et al. (25) used the BSCS in a 34-week longitudinal study and suggested that over the academic year, self-control was a predictor of autonomous motivation and controlled motivation. Although longitudinal research of self-control has become a hot topic, few studies have explored the measurement invariance of the instrument across different time points. Given that the BSCS is a self-report scale with subjectivity (26), individual's understanding of the measurement construct may change at different times. Thus, these conclusions in previous research might not be tenable if longitudinal measurement invariance (LMI) of the BSCS is not confirmed.

#### The Present Study

More and more scholars have begun to pay attention to the BSCS because of its brief set of items which can effectively measure the level of an individual's control abilities while greatly reducing the length of time and energy required of the participants. However, the factor structure of the scale remains controversial. In addition, no studies have been conducted as of yet to examine the psychometric property of the scale in Chinese adolescents.

More attention has focused on the long-term effects of self-control on the development of individual physical and mental health, in accordance with the dynamic development perspective. A fundamental assumption of a longitudinal study is the stability of the factor structure of measurement over time. In other words, the same scale should have the same meaning over all time points. However, considering the rapid development that occurs during adolescence, the meanings inherent in a scale might change at different age points (27). Thus, it is crucial to evaluate longitudinal measurement invariance in further longitudinal research. However, far fewer studies have examined the longitudinal measurement invariance of the BSCS up to now, particularly in Chinese adolescent populations.

In view of these points mentioned above, the primary objectives of the present study were to test the factor structure and evaluate the longitudinal measurement invariance of the BSCS in Chinese adolescents, with the aim of providing reference data for scholars in relevant fields.

## Methods

### Participants

Sample 1: Participants were 1,308 middle school students from four middle schools in Guiyang City, Guizhou Province, China. Their mean age was 14.71 years (*SD* = 1.79, age range = 12–19 years, 2.22% were missing data), and 50.38% were male (1.00% were missing data). Regarding their year of study, 15.83% were in grade seven, 20.72% in grade eight, 20.80% in grade nine, 17.28% in grade ten, 11.16% in grade eleven, 13.91% in grade twelve, and 0.30% of respondents were missing data. This batch of data was used mainly to verify the different factor structures of the BSCS, and proceed item analysis.

Sample 2: Participants were 942 middle school students from four middle schools in Zunyi City, Guizhou Province, China. Their mean age was 13.95 years (*SD* = 1.04, age range = 12–17, 4.14% were missing data), and 44.59% were male (2.55% were missing data). Regarding their year of study, 38.32% were in grade seven, 32.06% in grade eight, 27.07% in grade nine, and 2.55% were missing data. Due to the fact that our study took place at the same time as the Chinese National Matriculation Test, high school students were not included in this sample. This batch of data was used mainly to explore and verify the potential factor structure of the revised BSCS-12, and to evaluate the concurrent validity and internal consistency coefficient of the measurement.

Sample 3: Participants were 600 middle school students from a middle school in Guiyang City, Guizhou Province, China. The test-retest was performed 6 months later, and after eliminating invalid questionnaires, 496 (82.67%) valid paired questionnaires were obtained. Their mean age was 13.57 years (*SD* = 2.11, age range = 11–18, 1.21% did not specify), and 55.44% were male (0.81% sex unknown). This batch of data was used for test-retest reliability and longitudinal measurement invariance of the revised BSCS-12 (see Table 1).

**Table 1:** Three samples of data corresponding to different analysis procedure.

**Sample**		**Data analysis**			
Sample 1		CFA of the 13-item BSCS		Item analysis		
Sample 2	Sample 2a	EFA of the 12-item BSCS	PCA of the residuals	Criterion validity	Internal consistency
	sample 2b	CFA of the 12-item BSCS			
Sample 3		Test-retest reliability	Longitudinal measurement invariance		

*CFA, confirmatory factor analysis; EFA, exploratory factor analysis; PCA, principle component analysis*.

### Measures

#### Tangney's Brief Self-Control Scale

This scale was compiled by Tangney et al. (1), with 13 items in total. Responses were rated on a five-point Likert-type scale, ranging from 1 (very unlike me) to 5 (very like me), with nine items in reverse scoring. The higher the score, the stronger the self-control. The Chinese version of the BSCS was developed with a backtranslation procedure by two independent groups. The two groups consisted of eight psychology masters and one psychology PhD, respectively. Firstly, eight members of the first group independently translated the BSCS, and determined an initial Chinese version after discussing it together. Secondly, the original Chinese version of BSCS was translated back into English by another group. Then, a psychology master who had passed the Test for English Majors-Band 8 (TEM-8) compared the inconsistencies between the reverse translation version and the original English version. Finally, the final version of BSCS was obtained. In this study, the internal consistency coefficient of the scale was 0.79.

#### Rosenberg Self-Esteem Scale

This scale was revised by Ji et al. (28), with a total of 10 items. Responses were rated on a four-point Likert-type scale, ranging from 1 (very inconsistent) to 4 (very consistent). The higher the score, the higher the affirmation of their own value. In this study, the internal consistency coefficient of the scale was 0.85.

#### Social Anxiety Scale

This scale was revised by Scheier et al. (29), with a total of six items. Responses were rated on a four-point Likert-type scale, ranging from 1 (strongly disagree) to 4 (strongly agree). The higher the score, the higher the social anxiety. In this study, the internal consistency coefficient of this scale was 0.72.

#### Dickman-Impulsivity Inventory

This scale was prepared by Dickman (30), with a total of 23 items, each scored 1 point for the answer “yes” and 0 for “no.” The higher the score, the more impulsive the individual is. In this study, the internal consistency coefficient of this scale was 0.64.

### Procedures

Participants were invited to participate in the study during their free time in school. Considering that most of the participants in this study were under 18 years old, we informed the parents or guardians of the participants in advance through the school to obtain their consent. On the day the research took place, the four aforementioned questionnaires were completed under the supervision of the school's head teacher in order to avoid invalid questionnaires. In addition, to ensure the quality of the responses, the researchers told the participants that there was no right or wrong answer to any of the questionnaires and that they only needed to choose an answer that matched their status. Each participant completed the questionnaire independently and has given consent for their data to be used in the research. The study was approved by the Ethics Committee of Guizhou Normal University and the Ethical Approval Reference Number is 20191013.

### Data Analysis

The data was coded manually and entered into databases using EpiData 3.1, then converted to dta format. The relevant descriptive analysis, item analysis, exploratory factor analysis (EFA), concurrent validity, internal consistency coefficient, and the corresponding bootstrap statistical analysis were performed using STATA/SE 15.1. Mplus software version 8.3 was used to perform confirmatory factor analysis (CFA), and the Rasch analysis was run using WINSTEPS version 3.74.

As outlined through the research reviewed in the introduction, there are mainly two models of the BSCS: one and two-dimensional. To evaluate which model would perform best in our sample, CFA with the robust maximum likelihood (MLR) estimator was used to compare the factor structure of the various models since when response categories were fewer than five, maximum likelihood estimation was inappropriate (31). The parameters used for the fit indices were as follows: the chi-square, the Comparative Fit Index (CFI) (good ≥0.95; acceptable ≥0.90), the Tucker-Lewis index (TLI) (good ≥0.95; acceptable ≥0.90), the Root Mean Square Error of Approximation (RMSEA) (good ≤ 0.05; acceptable ≤ 0.08) (32).

The item-total correlation coefficients and inter-item correlation coefficients were used to evaluate the quality of items. An item-total correlations value ≥0.5 was considered to be satisfactory and a value ≥0.4 was considered to be acceptable, while an inter-item correlations value ≥0.3 was considered to be good and a value ≥0.2 was considered to be acceptable (33).

To explore and confirm the potential factor structure of the revised BSCS-12, Sample 2 was odd-even divided into two datasets (i.e., Sample 2a and Sample 2b). An EFA using the principal component factor (PCF) was conducted to extract factors. In EFA, uniqueness is the proportion of variance for the variable that is not explained by the common factors. It is often thought that variables are not well-explained by these factors when uniqueness is high. PCF is based on the assumption that the uniqueness is 0, which means there are no unique factors. Moreover, in factor analysis, the number of components to be extracted is that the average partial correlation of each variable is minimum after partialling out m principal components (34). Then minimum average partial correlation (MAP) was performed to supplement the accuracy of the eigenvalues when determining the number of factors, choosing a number of components at which the average squared partial correlations was minimum (34).

In CTT, the sum of all items is usually calculated directly into the total score. However, this is unreasonable since different items interpret different amounts of information about the underlying structure (35). In contrast, the Rasch model can be used to comprehensively assess the validity of the scale's underlying structure (36). Given that the structure of the BSCS was complicated and inconclusive in previous studies using classical test theory (CTT), Rasch analysis, which presumes that measurement error is different across individuals (37), was adopted on account of its superior performance in examining the underlying structure of an instrument robustly (38). The principle component analysis (PCA) of the residuals was performed to verify the unidimentionality assumption. An eigenvalue for the first contrast of residual lower than 2.0 or the proportion of explained variance by a measure >30% may indicate that the factor structure is unidimensional (39).

**Figure 1:**
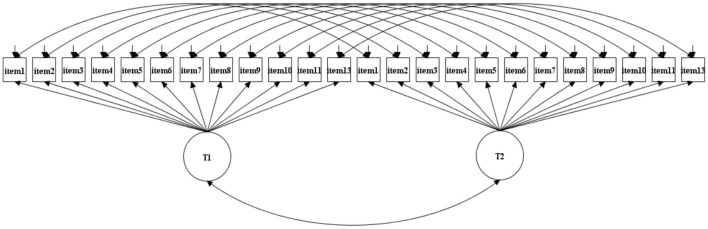
Model for the longitudinal measurement invariance. T1: BSCS-12 at time 1; T2: BSCS-12 at time 2.

Next, tests for LMI were performed with three nested models: (a) Configural invariance, which allows factor loadings and thresholds to be free (see Figure 1); (b) Metric invariance, which further constrains the factor loadings to be equal; (c) Scalar invariance, which further constrains item mean intercepts to be equal (40, 41). The fit of each subsequent model was compared to the previous model through the change in value of indices. The chi-square difference test was not used due to its sensitivity to a large sample size (42). Instead, it is considered that invariance exists if ΔRMSEA < 0.01 and ΔCFI ≤ −0.010 (40, 43).

Finally, to test the concurrent validity of the BSCS-12, the Pearson correlation coefficient was used and a level of significance of *p* ≤ 0.05 was adopted. Cronbach's alpha coefficient was calculated to assess internal consistency reliability (good ≥0.80; acceptable ≥0.70), and test-retest reliability was calculated to verify stability (very good ≥0.60; good ≥0.40) (44).

## Results

### Factor Structure of the BSCS

The fit indices of the six different models based on previous research are as follows: (1) Tangney model: χ^2^/≈370.161/65 (*p* < 0.001), CFI = 0.868, TLI = 0.841, RMSEA = 0.060 (0.054, 0.066); (2) Nebioglu model:χ^2^/≈139.815/26 (*p* < 0.001), CFI = 0.918, TLI = 0.887, RMSEA = 0.058 (0.049, 0.067); (3) Ferrari model: χ^2^/≈285.646/64 (*p* < 0.001), CFI = 0.904, TLI = 0.883, RMSEA = 0.051 (0.045, 0.058); (4) Maloney model: χ^2^/≈93.927/19 (*p* < 0.001), CFI = 0.930, TLI = 0.897, RMSEA = 0.055 (0.044, 0.066); (5) De Ridder model: χ^2^/≈229.783/34 (*p* < 0.001), CFI = 0.881, TLI = 0.842, RMSEA = 0.066 (0.058, 0.075); (6) Fung model: χ^2^/≈388.093/61 (*p* < 0.001), CFI = 0.858, TLI = 0.819, RMSEA = 0.064 (0.058, 0.070). According to all of these fit indices, the original unidimensional model (Tangney model) and the four-dimensional model (Fung model) did not show satisfactory model fit, with a lower value of both CFI and TLI. However, though the three two-dimensional models (Nebioglu, Ferrari, and Maloney models) showed varying degrees of improvement in CFI, they were all still unsatisfactory due to their unacceptable values for TLI.

Regarding the factor loadings of all of the items used in the different models (see Table 2), Item 12 (i.e., I refuse things that are bad for me) consistently showed a very low value: 0.157 in the Tangney model, 0.289 in the Ferrari model, and 0.149 in the De Ridder model. It is worth noting that other research has also found poor value of factor loading (value < 0.30) (19) and cross-loading (18) in the EFA of Item 12. This finding reminded us that an analysis of item quality is important.

**Table 2:** Standardized Factor Loadings for the different models.

**Item**		**Standardized factor loadings**												
		**Tangney**	**Nebioglu**		**Ferrari**		**Maloney**		**De Ridder**		**Fung**			
		**model**	**model**		**model**		**model**		**model**		**model**			
		**BSCS**	**SD**	**IM**	**SD**	**IC**	**RE**	**IM**	**INH**	**INI**	**SD**	**IM**	**HH**	**SR**
Item 1	I am good at resisting temptation	0.399	0.416	–	–	0.526	0.419	–	0.390	–	–	0.516	–	–
Item 2*	I have a hard time breaking bad habits	0.530	0.539	–	0.528	–	0.528	–	0.535	–	–	–	0.590	–
Item 3*	I am lazy	0.634	–	–	0.633	–	–	–	–	0.614	–	–	0.710	–
Item 4*	I say inappropriate things	0.524	–	–	0.527	–	–	–	–	–	0.548	–	–	–
Item 5*	I do certain things that are bad for me, if they are fun	0.514	–	0.504	0.520	–	–	0.531	0.478	–	0.542	–	–	–
Item 6*	I wish I had more self-discipline	0.398	0.421	–	0.400	–	0.415	–	–	–	–	–	–	–
Item 7*	Pleasure and fun sometimes keep me from getting work done	0.491	–	0.519	0.501	–	–	0.472	0.480	–	–	–	–	0.557
Item 8*	I have trouble concentrating	0.575	–	0.588	0.578	–	–	–	–	0.593	–	–	–	0.692
Item 9	I am able to work effectively toward long-term goals	0.339	–	–	–	0.483	–	–	–	0.324	–	0.460	–	0-
Item 10*	Sometimes I can't stop myself from doing something, even if I know it is wrong	0.605	–	0.648	0.614	–	–	0.683	0.601	–	0.675	–	–	–
Item 11*	I often act without thinking through all the alternatives	0.549	–	0.584	0.556	–	–	0.599	–	0.547	0.607	–	–	–
Item 12	I refuse things that are bad for me	0.157	–	–	–	0.289	–	–	0.149	–	–	–	–	–
Item 13	People would say that I have iron self- discipline	0.441	0.463	–	–	0.548	0.483	–	–	–	–	0.536	–	–

* *Recoded items of the BSCS; BSCS, Brief Self-Control Scale; SD, Self-Discipline; IM, Impulsivity; IC, Impulse Control; RE, Restraint; INH, Inhibition; INI, Initiation; HH, healthy habits; SR, self-regulation*.

### Item Analysis of the BSCS

According to the results of the CFA, we decided to further evaluate the item quality. The item-total correlation coefficients and item-item correlation coefficients are shown in Table 3. The item-total correlations of all items, except for Item 12, reached the acceptable criteria of 0.4. As for item-item correlations, Item 12 still had an unsatisfactory score, ranging from −0.02 to 0.21. Thus, considering the poor factor loading and item quality, we decided to drop Item 12 and retain the other 12 items for subsequent validity and reliability analysis.

**Table 3:** Correlation coefficients of all items of the BSCS.

**Item**	**Item-total correlation**	**Item-item correlation**												
		**1**	**2**	**3**	**4**	**5**	**6**	**7**	**8**	**9**	**10**	**11**	**12**	**13**
Item 1	0.48	–												
Item 2	0.57	0.25	–											
Item 3	0.65	0.24	0.42	–										
Item 4	0.57	0.21	0.25	0.34	–									
Item 5	0.58	0.17	0.25	0.30	0.35	–								
Item 6	0.47	0.12	0.19	0.25	0.22	0.25	–							
Item 7	0.54	0.13	0.24	0.28	0.24	025	0.33	–						
Item 8	0.61	0.24	0.33	0.38	0.22	0.24	0.23	0.39	–					
Item 9	0.44	0.28	0.18	0.23	0.18	0.16	0.08	0.14	0.22	–				
Item 10	0.63	0.23	0.26	0.37	0.33	0.37	0.19	0.30	0.35	0.11	–			
Item 11	0.58	0.19	0.28	0.31	0.32	0.28	0.16	0.26	0.32	0.13	0.45	–		
Item 12	0.29	0.17	0.08	0.08	0.06	0.12	0.02	−0.02	0.10	0.21	0.08	0.02	–	
Item 13	0.52	0.25	0.24	0.30	0.22	0.18	0.22	0.17	0.21	0.24	0.25	0.23	0.17	–

*The order of all items is consistent with Table 2*.

### EFA of the BSCS-12

After dropping Item 12, EFA was applied to the remaining items to determine the underlying structure of the BSCS-12 based on Sample 2a (*n* = 471). The results yielded two factors with Kaiser eigenvalues >1.0, and the explained variance of Factor 1 and Factor 2 were 33.2 and 9.9%, respectively. However, given that their ratio was >3 (3.978/1.186 >3.354), it was better to retain one factor (45). Additionally, the results of minimum average partial correlations (MAP) suggested one principal component should be extracted due to its minimum average squared partial correlation value of 0.015. To sum up, then, the potential structure of BSCS-12 is one-dimensional.

### CFA of the BSCS-12

Guided by the results of the EFA, the one-dimensional model of the BSCS-12 was tested using Sample 2b (*n* = 471). CFA was conducted and the fit indices of the unidimensional model, were as follows: χ^2^/≈105.858/54χ^2^/≈1.960 (*p* < 0.001) CFI = 0.939, TLI = 0.925, RMSEA = 0.045 (0.032, 0.058). According to these values, the fit indices for the model were acceptable, indicating that the BSCS-12 was a one-dimensional structure in our Chinese adolescent sample.

### Principal Component Analysis of the Residuals

As a unidimensional model of the BSCS-12 was generated based on the results of EFA and CFA, a principal component analysis (PCA) of the residuals was performed to provide more sufficient evidence for the assumption of unidimensionality (see Table 4). The proportion of raw variance explained by the measures was 35.2% and the raw variance unexplained in the first contrast was 1.5, indicating that the unidimensional assumption of the BSCS-12 was reasonable.

**Table 4:** Variance of standardized residuals for the BSCS-12.

	**Eigenvalues**	**Observed (%)**	**Expected (%)**
**Goal setting**			
Total raw variance =	18.5	100.0	100.0
Raw variance explained by measures =	6.5	35.2	35.4
Raw variance explained by persons =	2.0	10.7	10.8
Raw variance explained by items =	4.5	24.5	24.6
Raw unexplained variance (total) =	12.0	64.8	64.6
Raw variance unexplained in 1st contrast =	1.5	8.4	12.9

### Longitudinal Measurement Invariance

The results of the three nested models (i.e., configural, metric, and scalar models) are located in Table 5. Although the chi-square test was significant, the other indices yielded acceptable model fit (CFI = 0.925, TLI = 0.913, RMSEA = 0.039), indicating that configural invariance was held and that further inter-model assessment could proceed. The comparison between the metric and configural models produced low differences in indices (ΔCFI = −0.008, ΔRMSEA = 0.001), with similar results in the comparison between the scalar and metric models (ΔCFI = −0.010, ΔRMSEA = 0.001). These findings supported the longitudinal measurement invariance for the BSCS-12 in Chinese adolescents.

**Table 5:** CFA of the BSCS and LMI of the BSCS-12 over a 6-month time period.

**Model**		**Model fit**						**Difference in model fit**	
		** χ^2^**	***df***	**CFI**	**TLI**	**RMSEA**	**90% CI**	**ΔCFI**	**ΔRMSEA**
Tangney model		370.161***	65	0.868	0.841	0.060	(0.054 0.066)	–	–
Nebioglu model		139.815***	26	0.918	0.887	0.058	(0.049 0.067)	–	–
Ferrari model		285.646***	64	0.904	0.883	0.051	(0.045 0.058)	–	–
Maloney model		93.927***	19	0.930	0.897	0.055	(0.044 0.066)	–	–
De Ridder model		229.783***	34	0.881	0.842	0.066	(0.058 0.075)	–	–
Fung model		388.093***	61	0.858	0.819	0.064	(0.058 0.070)	–	–
BSCS-12		105.858***	54	0.939	0.925	0.045	(0.032 0.058)	–	–
LMI model	Configural	415.633*	239	0.925	0.913	0.039	(0.032 0.045)	–	–
	Metric	444.299*	250	0.917	0.908	0.040	(0.033 0.045)	−0.008	0.001
	Scalar	479.104*	261	0.907	0.902	0.041	(0.035 0.047)	−0.010	0.001

*CFA, Confirmatory factor analysis; LMI, longitudinal measurement invariance*;

* *p < 0.05*,

*** *p < 0.001. df, degrees of freedom; CFI, comparative fit index; TLI, Tucker–Lewis index; RMSEA, root mean square error of approximation; 90% CI, RMSEA 90% confidence interval*.

### Reliability and Concurrent Validity

The BSCS-12 presented adequate internal consistency (α = 0.81) based on Sample 2 results. The test-retest reliability was 0.70 based on Sample 3 results, which was very good for the revised scale and indicated that the 12-item scale showed satisfactory test-retest reliability.

Based on the original study by Tangney et al. (1), the RSES, SAS, and DII were also selected as criteria in our study. The correlation coefficients of the BSCS and the RSES, SAS, and DII were 0.489, −0.234, and −0.485, respectively (all *p'*s < 0.001).

## Discussion

The BSCS (1) is a good instrument consisting of only 13 items, used to measure the level of an individual's trait self-control. It shows good reliability and validity, and has more advantages than its original version, which contains more items and dimensions and could lead to response fatigue in subjects, which would then interfere with the accuracy of assessment results. However, the factor structure of the BSCS has remained ambiguous and until now, has only attracted less attention from Chinese scholars. Additionally, given the frequency of juvenile delinquency, and a meta-analysis of general crime theory that shows that self-control is one of the most important factors related to criminal behavior (46), it is crucial to pay attention to self-control in adolescents. In view of this, three surveys were conducted for this study to collect data to validate the BSCS using three Chinese adolescent samples.

The most important contribution of this study is to reevaluate the factor structure of BSCS in the Chinese population and propose a new 12-item version. In addition, the stability of the 12-item version across time was investigated, which provides reliable data for future longitudinal studies. Based on previous studies, different factor models (one-factor, two-factor and four-factor models) of the BSCS were verified. Though several two-dimensional models showed better model fit compared with the unidimensional model, none of them were acceptable according to goodness-of-fit guidelines. The better performance of the two-dimensional models may be because of the expurgation of items from the BSCS (47). At the same time, this study did not support the 4-factor model proposed by Fung et al. (20).

It's worth noting that poor factor loadings of item 12 (i.e., I refuse things that are bad for me) were found in both the one-dimensional or two-dimensional CFA models, which is consistent with most previous studies (18–20). This may be a partial reason for the lower model fit when performing CFA. After analyzing the description of item 12, we found that it was more a description of an individual's instincts about “seeking profits while avoiding harm” than a decision that requires an individual to make through a cognitive process. In other words, this item may not be consistent with the original concept of self-control. Meanwhile, the results of item-total correlation and inter-item correlation coefficients also showed that all BSCS items had a high intrinsic correlation, with the exception of Item 12. Thus, item 12 was eventually removed.

After dropping item 12, we reassessed the factor structure of 12-item BSCS. According to the results of the EFA, the measure can be considered to be a one-dimensional structure. Then the one-dimensional model was used for CFA, and results indicated that all fitting indices of the model satisfied the measurement criteria. Meanwhile, considering the inherent limitations of CTT, the Rasch analysis was also used, and the result of the PCA indicated that the initial assumption of unidimensionality was valid. Therefore, consistent with the original research, it can be considered that the revised BSCS-12 is also a one-dimensional structural scale.

The internal consistency coefficient of BSCS-13 was 0.79, but after removing item 12, the internal consistency coefficient of BSCS-12 increased to 0.81, replicating high values in previous studies (1, 20). In addition, the test-retest reliability of the scale was also satisfactory, indicating that the revised BSCS-12 has good reliability. In accordance with the original study (1), the RSES, SAS, and DII were selected as comparison criteria. The results showed that there was a significant positive correlation between the BSCS-12 and the RSES, a significant negative correlation with the SAS, and a moderate negative correlation with the DII, which was consistent with the findings of the original scale. In general, the revised BSCS-12 has good concurrent validity.

Finally, a longitudinal measurement invariance was evaluated for BSCS-12 to ensure the inferences of validity. Three progressively more stringent models were estimated and compared. Given that all fit indices differences between models were negligible and met criteria for acceptability, longitudinal metric and scalar invariance for the BSCS-12 were upheld. In other words, the BSCS-12 has a good construct validity, and its format holds the same function and meaning for Chinese adolescents over time.

## Conclusion

The revised BSCS-12 is a one-dimensional scale that has good psychometric properties among Chinese adolescents, effectively measuring the level of an individual's ability in self-control.

### Limitations

The present study was the first attempt to validate the BSCS for Chinese adolescents. Whereas, some meaningful findings were yielded to in this study, several limitations should also be noted. First and foremost, while Item 12 was removed according to statistical analysis used in the present study, this conclusion should be taken with caution, given that all three samples in this study were from the same geographic area, which may have affected variability and limited the generalization performance of the revised scale in other regions. Additionally, although this study yielded a significant result of longitudinal measurement invariance for the revised 12-item BSCS, these findings may only apply to middle school students. In the future, more studies are needed to verify these conclusions in other groups.

## Data Availability Statement

The original contributions presented in the study are included in the article/Supplementary Files, further inquiries can be directed to the corresponding author/s.

## Ethics Statement

The studies involving human participants were reviewed and approved by Ethics Committee of Guizhou Normal University. Written informed consent to participate in this study was provided by the participants' legal guardian/next of kin.

## Author Contributions

WC concepted the article and provided framework of the manuscript. GZ and XT analyzed the data and drafted the manuscript. The final version was approved by SZ. All authors contributed to the article and approved the submitted version.

## Funding

This study was funded by the Ministry of Education Humanities and Social Sciences Research Youth Fund Project (Grant No. 18XJC190001), Natural Science Research Funding Project of the Department of Education of Guizhou Province (Grant No. Qian Ke He KY Zi [2021]299), Guizhou Educational Reform and Development Research Major Project (ZD202009), Phased Achievements of Guizhou Province Philosophy and Social Science Planning Project (21GZZD45), and Humanities and Social Science Research Project of Higher Education Institutions of Guizhou Provincial Department of Education (2022ZD006).

## Conflict of Interest

The authors declare that the research was conducted in the absence of any commercial or financial relationships that could be construed as a potential conflict of interest.

## Publisher's Note

All claims expressed in this article are solely those of the authors and do not necessarily represent those of their affiliated organizations, or those of the publisher, the editors and the reviewers. Any product that may be evaluated in this article, or claim that may be made by its manufacturer, is not guaranteed or endorsed by the publisher.

## References

[1] Tangney JP Baumeister RF Boone AL. High self-control predicts good adjustment, less pathology, better grades, and interpersonal success. J Person. (2004) 72:271–324. 10.1111/j.0022-3506.2004.00263.x 15016066

[2] Woessner G Schneider S. The role of self-control and self-esteem and the impact of early risk factors among violent offenders. Crim Behav Mental Health. (2013) 23:99–112. 10.1002/cbm.1863 23595861

[3] Englert C Bertrams A. Too exhausted for operation? Anxiety, depleted self-control strength, perceptual-motor performance. Self Identity. (2013) 12:650–62. 10.1080/15298868.2012.718865

[4] De Ridder DTD Lensvelt-Mulders G Finkenauer C Stok FM Baumeister RF. Taking stock of self-control: a meta-analysis of how trait self-control relates to a wide range of behaviors. Person Soc Psychol Rev. (2012) 16:76–99. 10.1177/1088868311418749 21878607

[5] Finkel EJ Campbell WK. Self-control and accommodation in close relationships: an interdependence analysis. J Person Soc Psychol. (2001) 81:263–77. 10.1037/0022-3514.81.2.263 11519931

[6] Malouf ET Schaefer KE Witt EA Moore KE Stuewig J Tangney JP. The brief self-control scale predicts jail inmates' recidivism, substance dependence, post-release adjustment. Person Soc Psychol Bull. (2014) 40:334–47. 10.1177/0146167213511666 24345712 PMC4485378

[7] Carver CS Sinclair S Johnson SL. Authentic and hubristic pride: differential relations to aspects of goal regulation, affect, and self-control. J Res Person. (2010) 44:698–703. 10.1016/j.jrp.2010.09.004 21769159 PMC3137237

[8] De Kemp RA Vermulst AA Finkenauer C. Self-control and early adolescent antisocial behavior: a longitudinal analysis. J Early Adol. (2009) 29:497–517. 10.1177/0272431608324474

[9] Moffitt TE Arseneault L Belsky D Dickson N Hancox RJ Harrington HL.. A gradient of childhood self-control predicts health, wealth, public safety. Proc Natl Acad Sci USA. (2011) 108:2693–8. 10.2307/41002200 21262822 PMC3041102

[10] De Vries RE Van Gelder JL. Tales of two self-control scales: relations with five-factor and HEXACO traits. Person Ind Differen. (2013) 54:756–60. 10.1016/j.paid.2012.12.023

[11] Frick PJ Lahey BB Loeber R. Oppositional defiant disorder and conduct disorder: a meta-analytic review of factor analyses and cross-validation in a clinic sample. Clin Psychol Rev. (1993) 13:319–40. 10.1016/0272-7358(93)90016-F

[12] Tan SH Guo YY. Revision of self-control scale for chinese college students. Chin J Clin Psychol. (2008) 16:468–70. 10.16128/j.cnki.1005-3611.2008.05.022

[13] Unger A Bi CZ Xiao YY Ybarra O. The revising of the tangney self-control scale for chinese students. PsyCh J. (2016) 5:101–17. 10.1002/pchj.128 27144924

[14] Bertrams A Dickhauser O. Measuring dispositional self-control capacity: a german adaptation of the short form of the self-control scale (SCS-K-D). Diagnostica. (2009) 55:2–10. 10.1026/0012-1924.55.1.2

[15] Brevers D Foucart J Verbanck P Turel O. Examination of the validity and reliability of the french version of the brief self-control scale. Can J Behav Sci. (2017) 49:1–16. 10.1037/cbs0000086 29200467 PMC5707128

[16] De Ridder DTD De Boer BJ Lugtig P Bakker AB Van Hooft EAJ. Not doing bad things is not equivalent to doing the right thing: distinguishing between inhibitory and initiatory self-control. Person Ind Differen. (2011) 50:1006–11. 10.1016/j.paid.2011.01.015

[17] Ferrari JR Stevens EB Jason LA. The role of self-regulation in abstinence maintenance: effects of communal living on self-regulation. J Groups Addict Rec. (2009) 4:32–41. 10.1080/15560350802712371 20689650 PMC2916178

[18] Maloney PW Grawitch MJ Barber LK. The multi-factor structure of the brief self-control scale: discriminant validity of restraint and impulsivity. J Res Person. (2012) 46:111–5. 10.1016/j.jrp.2011.10.001

[19] Nebioglu M Konuk N Akbaba S Eroglu Y. The investigation of validity and reliability of the Turkish version of the brief self-control scale. Klinik Psikofarmakoloji Bülteni Bull Clin Psychopharmacol. (2012) 22:340–51. 10.5455/bcp.20120911042732

[20] Fung S Kong CYW Huang Q. Evaluating the dimensionality and psychometric properties of the brief self-control scale amongst Chinese University students. Front Psychol. (2020) 10:2903. 10.3389/fpsyg.2019.02903 31969852 PMC6960225

[21] Gottfredson MR Hirschi T. A general Theory of Crime. New York, NY: Stanford University Press (1990).

[22] Heatherton T Tice DM. Losing Control: How and Why People Fail at Self-Regulation. San Diego, CA: Academic Press, Inc. (1994).

[23] Johnson W McGue M Krueger RF. Personality stability in late adulthood: a behavioral genetic analysis. J Person. (2005) 73:523–52. 10.1111/j.1467-6494.2005.00319.x 15745440

[24] Ng-Knight T Shelton KH Riglin L McManus IC Frederickson N Rice F. A longitudinal study of self-control at the transition to secondary school: considering the role of pubertal status and parenting. J Adol. (2016) 50:44–55. 10.1016/j.adolescence.2016.04.006 27183536

[25] Holding A Hope N Verner-Filion J Koestner R. In good time: a longitudinal investigation of trait self-control in determining changes in motivation quality. Person Ind Differen. (2019) 139:132–7. 10.1016/j.paid.2018.11.001

[26] Borsboom D. Measuring the Mind: Conceptual Issues in Contemporary Psychometrics. New York, NY: Cambridge University Press (2005).

[27] Liu Y Millsap RE West SG Tein J-Y Tanaka R. Testing measurement invariance in longitudinal data with ordered-categorical measures. Psychol Methods. (2017) 22:486–506. 10.1037/met0000075 27213981 PMC5121102

[28] Ji FY Yu X. Self-esteem scale. Chin Mental Health J. (1999) 13:318–20.

[29] Scheier MF Carver CS. The self-consciousness scale: a revised version for use with general populations. J Appl Soc Psychol. (1985) 15:687–99. 10.1111/j.1559-1816.1985.tb02268.x

[30] Dickman SJ. Functional and dysfunctional impulsivity: personality and cognitive correlates. J Person Soc Psychol. (2001) 21:95–102. 10.1037/0022-3514.58.1.95 2308076

[31] DiStefano C. The impact of categorization with confirmatory factor analysis. Struct Equat Mod. (2002) 9:327–46. 10.1207/S15328007SEM0903_2

[32] Li MS Wang MC Shou YY Zhong CX Ren F Zhang XT. Psychometric properties and measurement invariance of the brief symptom inventory-18 among Chinese insurance employees. Front Psychol. (2018) 9:519–26. 10.3389/fpsyg.2018.00519 29720953 PMC5915545

[33] Silva-Rocha VV de Sousa DA Osório FL. Psychometric properties of the brazilian version of the sport anxiety scale-2. Front Psychol. (2019) 10:806–14. 10.3389/fpsyg.2019.00806 31040807 PMC6477035

[34] Velicer W. Determining the number of components from the matrix of partial correlations. Psychometrika. (1976) 41:321–7. 10.1007/BF02293557

[35] Mitchell-Parker K Medvedev ON Krägeloh CU Siegert RJ. Rasch analysis of the frost multidimensional perfectionism scale. Austr J Psychol. (2017) 70:258–68. 10.1111/ajpy.12192

[36] Chen W Zhang G Tian X Wang L Luo J. Rasch analysis of work-family conflict scale among chinese prison police. Front Psychol. (2021) 12:537005. 10.3389/fpsyg.2021.537005 34025488 PMC8136239

[37] Van Zile-Tamsen C. Using rasch analysis to inform rating scale development. Res Higher Educ. (2017) 58:922–33. 10.1007/s11162-017-9448-0

[38] Rustøen T Lerdal A Gay C Kottorp A. Rasch analysis of the herth hope index in cancer patients. Health Quality Life Outcomes.. (2018) 16:196–205. 10.1186/s12955-018-1025-5 30285767 PMC6171309

[39] Pichardo MC Cano F Garzón-Umerenkova A de la Fuente J Peralta-Sánchez FJ Amate-Romera J. Self-regulation questionnaire (SRQ) in spanish adolescents: factor structure and rasch analysis. Front Psychol. (2018) 9:1370–83. 10.3389/fpsyg.2018.01370 30147667 PMC6095962

[40] Gross TJ Fleming CB Mason WA Haggerty KP. Alabama parenting questionnaire−9: longitudinal measurement invariance across parents and youth during the transition to high school. Assessment. (2015) 24:646–59. 10.1177/1073191115620839 26671892 PMC4909593

[41] Motl RW McAuley E Mullen S. Longitudinal measurement invariance of the multiple sclerosis walking scale-12. J Neurol Sci. (2011) 305:75–9. 10.1016/j.jns.2011.03.008 21474149

[42] Wu PC. Longitudinal measurement invariance of beck depression inventory–ii in early adolescents. Assessment. (2017) 24:337–45. 10.1177/1073191115608941 26423351

[43] Esnaola I Benito M Antonio-Agirre I Axpe I Lorenzo M. Longitudinal measurement invariance of the satisfaction with life scale in adolescence. Quality Life Res. (2019) 28:2831–7. 10.1007/s11136-019-02224-7 31177412

[44] Wang MC Colins OF Deng QW Andershed H Deng JX Ye HS. Psychometric properties of the original and shortened version of the Youth psychopathic traits inventory among Chinese adolescents. J Psychopathol Behav Assess. (2017) 39:620–34. 10.1007/s10862-017-9619-5

[45] Bond T Fox CM. Applying the Rasch Model: Fundamental Measurement in the Human Sciences 3 ed. New York, NY: Routledge (2015).

[46] Pratt TC Cullen FT. The empirical status of gottfredson and Hirschi's general theory of crime: a meta-analysis. Criminology. (2010) 38:931–64. 10.1111/j.1745-9125.2000.tb00911.x

[47] Lindner C Nagy G Retelsdorf J. The dimensionality of the brief self-control scale—an evaluation of unidimensional and multidimensional applications. Person Ind Differen. (2015) 86:465–73. 10.1016/j.paid.2015.07.006

